# Cystic fibrosis-related diabetes is associated with reduced airway microbial diversity

**DOI:** 10.1186/s12931-026-03598-2

**Published:** 2026-02-27

**Authors:** Stefanie Diemer, Katja Kozjek, Lisa I. Påhlman

**Affiliations:** 1 Department of Paediatrics, Skåne University Hospital, Lund, Sweden; 2 Division of Infection Medicine, Department of Clinical Science Lund, Lund University, BMC B14, Lund, SE-221 84 Sweden; 3 Wallenberg Centre for Molecular Medicine, Lund, Sweden; 4 Department of Laboratory Medicine, National Bioinformatics Infrastructure Sweden (NBIS), Lund University, SciLifeLab, Lund, Sweden; 5 Department of Infectious Diseases, Skåne University Hospital, Lund, Sweden

**Keywords:** Cystic fibrosis, Cystic fibrosis-related diabetes, Airway inflammation, Lung function, Microbiome

## Abstract

**Background:**

Cystic fibrosis (CF) is a genetic disorder characterized by chronic airway inflammation and lung function decline. CF-related diabetes (CFRD) is the main extrapulmonary complication and it is tightly linked to an impaired lung function, but the underlying mechanisms behind these observations are incompletely understood. In the present study, we aimed to compare airway microbiome compositions between pwCF with and without CFRD.

**Methods:**

Sputum samples from pwCF with and without CFRD were analysed for inflammatory cytokines using MesoScale assays and total bacterial load using quantitative PCR of the 16s rRNA gene. Bacterial sputum microbiomes were analysed with 16s rRNA sequencing and characterized based on richness and evenness. Bray-Curtis was used to determine the distance in microbiome compositions between samples.

**Results:**

Forty-four pwCF were included, of which 59% were diagnosed with CFRD. The CFRD group had significantly lower lung function and elevated sputum levels of IL-1β compared to pwCF without CFRD. The CFRD sputum microbiome was characterized by reduced bacterial diversity, but this association was attenuated after adjusting for lung function. The distance in microbiome composition did not differ between groups. *Abiotrophia*, *Anaeroglobus* and *Escherichia-Shigella* were significantly enriched in the CFRD group, while *Neisseria*, *Prevotella* and *Streptococcus* were more abundant in pwCF without CFRD.

**Conclusion:**

CFRD is associated with impaired lung function, elevated airway inflammation, and a sputum microbiome characterized by reduced bacterial diversity and a more dysbiotic composition. The decreased microbial diversity observed in pwCF with CFRD was predominantly driven by impaired lung function rather than by CFRD itself.

## Introduction

Cystic fibrosis (CF) is a genetic disorder caused by mutations in the *CF transmembrane conductance regulator* (*CFTR*) gene [1], which encodes a chloride and bicarbonate ion channel. The CFTR protein is primarily expressed in epithelial cells of exocrine glands including the airways, pancreas and gastrointestinal tract. In the airways, defective CFTR function leads to dehydrated and viscous mucus followed by impaired mucociliary clearance. This dysfunction facilitates microbial colonisation and persistent inflammation, resulting in progressive pulmonary disease and declining lung function.

CF-related diabetes (CFRD) is the most common extrapulmonary complication of CF and is associated with increased morbidity and mortality [2]. CFRD affects approximately 30% of pwCF, with prevalence increasing with age [3, 4]. There is a strong association between CFRD and reduced lung function [5, 6], although the underlying mechanisms remain incompletely understood. These are likely multifactorial and believed to involve inflammatory pathways [7, 8].

The human airways harbour a diverse and dynamic microbial community that plays a critical role in maintaining respiratory health. In healthy individuals, the airway microbiota is characterized by a relatively stable and diverse composition, which contributes to immune homeostasis and resistance to pathogen overgrowth [9]. In contrast, respiratory diseases including asthma, chronic obstructive pulmonary disease and CF have been associated with airway dysbiosis, defined as an imbalance in the composition or function of microbial communities [9, 10]. In CF, airway dysbiosis is marked by reduced microbial diversity and the dominance of pathogens such as *Pseudomonas aeruginosa* and *Staphylococcus aureus*. This shift in microbial ecology is associated with age, persistent infection, increased inflammation, and accelerated lung function decline [11].

Emerging evidence suggests that hyperglycaemia may influence airway microbial dynamics [12]. Glucose serves as a preferred carbon source for many bacterial species, and elevated glucose levels enhance virulence traits related to chronic infection, such as adhesion and biofilm formation [13]. Compared to healthy individuals, pwCF exhibit elevated airway glucose concentrations with even higher concentrations observed in those with CFRD [14]. Individuals with CFRD have higher odds of being chronically infected with *P. aeruginosa* [15] and a murine model demonstrated that diabetic conditions resulted in increased bacterial proliferation and more severe infection [16]. Collectively, these findings suggest that hyperglycaemia may influence microbial communities and contribute to airway dysbiosis, potentially accelerating lung function decline in CFRD.

We recently reported that CFRD is associated with increased airway inflammation and distinct sputum proteome profiles [17]. In this follow-up study, we aimed to characterize the sputum microbiome in pwCF with and without CFRD. We hypothesized that dysregulated glucose levels may influence the composition of airway bacterial communities, thereby modulate inflammatory responses and contribute to disease progression.

## Methods

### Study population

All study participants were prospectively recruited at the CF centre of Skåne University Hospital Lund, Sweden, during 2021 and 2022. Inclusion criteria were age ≥ 18 years, a typical clinical presentation of CF lung disease and diagnosed disease-causing mutations in the *CFTR* gene. Exclusion criteria were a history of organ transplantation, inability to expectorate a sputum sample or ongoing airway exacerbation.

Individuals without a known CFRD diagnosis underwent regular oral glucose tolerance test (OGTT) screening according to international guidelines. CFRD was defined as a fasting blood glucose ≥ 7 mmol/l and/or a 120-minute glucose value ≥ 11,1 mmol/l during OGTT [18].

Clinical data were collected from the Swedish CF registry. Forced expiratory volume in 1 s in percent of predicted (FEV_1_pp) was measured using the Global Lung Function Initiative Eq [19]. Chronic infection with *P. aeruginosa* was defined according to Leeds criteria [20].

The study was approved by the Swedish Ethical Review Authority, Uppsala, Sweden (reference number 2018/54 with amendments 2021-05475-02 and 2023-06075-02 and reference number 2021 − 01191). Written informed consent was obtained from all participants.

### Sputum sample collection and preparation

Study participants donated an expectorated or induced sputum sample to the study at the time of their annual clinical review. The sputum sample collection was supervised by a physiotherapist.

Sputum samples were liquefied with 0,1% dithiothreitol (DTT) (Sigma-Aldrich) as described before [21]. One aliquot of the liquefied sputum was stored at -80 °C for later DNA extraction. The remaining sample was centrifuged at 1000 x g for 10 min, and the cell-free supernatant was collected and stored at − 80 °C until further analysis.

### Nucleic acid extraction and quantitative PCR

Total DNA was extracted from 200 µl of liquefied sputum using bead-beating with Pathogen Lysis Tubes S (Qiagen ref.19091) followed by QIAamp UCP Pathogen Mini Kit (Qiagen ref.50214) according to the protocol of the manufacturer. Total bacterial load was assessed with quantitative PCR of the bacterial 16s rRNA gene using primers described previously [22]. Sputum samples were diluted 1:1000 in nuclease-free H_2_O and 5 µl template was added to 15 µl mastermix containing iTaq Universal SYBRGreen Supermix (Bio-Rad), primers and nuclease free H_2_O. Known concentrations of *P. aeruginosa* DNA in 10-fold dilutions were analysed in parallel and used as a standard.

### Quantification of inflammatory cytokines

Cytokine levels in cell-free sputum samples were analysed using Mesoscale immunoassays (Mesoscale Diagnostics LLA., Maryland, USA) according to the protocol provided by the manufacturer. IL-6 and IL-1β were analysed in a multiplex U-PLEX with a sample dilution of 1:5. All samples were analysed in duplicates.

### Statistical analysis

Statistical analyses were performed using GraphPad Prism 10.0.2 software (GraphPad Software, San Diego, CA). Comparisons between groups were made using Mann-Whitney U test for continuous variables and Chi square test for categorical values. Two-tailed *p* ≤ 0.05 was determined as statistically significant.

### Sequencing

Total sputum DNA, extracted as described above, was used as template. Amplicon libraries targeting the hypervariable V3-V4 region of the 16s rRNA gene were prepared using the 515 F/806R primer pair. Library preparation and sequencing were performed by the Center for Translational Genomics (CTG), Lund University, Lund, Sweden, on the Illumina NextSeq 2000 platform (Illumina, USA). The raw sequencing data (fastq files) is stored and publicly available in the European Nucleotide Archive (ENA) database (http://www.ebi.ac.uk) under the project ID PRJEB102706.

### Microbiome analysis – bioinformatics and statistical analyses

Sequencing data was processed using the nf-core/ampliseq pipeline (version 2.9.0) [23] of the nf-core collection of workflows [24], utilising reproducible software environments from the Bioconda [25] and Biocontainers [26] projects. Quality of sequencing reads was checked using the FastQC (https://www.bioinformatics.babraham.ac.uk/projects/fastqc/) and results were summarized with MultiQC [27]. Further, Cutadapt was used to trim primers and all untrimmed sequences were discarded [28]. The amplicon sequence variants (ASVs) were generated from the sequences free of primers and adapters for each sample independently using DADA2 [29]. The taxonomic classification of curated ASVs was performed using the DADA2 in combination with the Silva 138.1 prokaryotic SSU database [30].

All microbiome related statistical analyses were performed using the R statistical software (version 4.1.1). Prior alpha- and beta-diversity analyses, ASVs were agglomerated at genus level. Alpha-diversity was estimated using the number of observed genera (richness, how many genera there are) and Shannon index (diversity, how many genera and their distribution) [31]. These two diversity indices were computed using the ‘estimate_richness’ from the ‘phyloseq’ package [32]. Normal distribution was assessed using the Shapiro–Wilk test. To compare the alpha diversity of the pwCF with and without CFRD, standard t-test was used.

Univariate linear regression was performed to evaluate the association between CFRD and Shannon diversity. To account for lung function, we subsequently conducted multiple linear regression with the Shannon diversity index as the dependent variable and CFRD status (binary: yes/no) and FEV1pp predicted (continuous) as independent variables. All regression analyses were performed using SPSS version 30.0 (IBM SPSS Ing., Chicago, IL, USA).

The beta-diversity was estimated using the Bray-Curtis distance and visualized by principal coordinate analysis (PCoA). To determine whether the differences in the airway microbiome were driven by CFRD a permutational multivariate analysis of variance (PERMANOVA) using the ‘adonis2’ function from the ‘vegan’ package [33] was performed. The relationship between clinical parameters and the airway microbiome community composition was assessed using the ‘envfit’ function from the ‘vegan’ package [33]. The significant variables (*p* < 0.05) were fitted onto the PCoA ordination as vectors (represented by arrows) and dots are colored according to the diagnosis group. Differential abundance analysis using DESeq2 [34] was performed to identify differentially abundant microorganisms in pwCFRD and pwCF.

All scripts used for the bioinformatics and statistics analyses are openly available at the following repository: https://github.com/NBISweden/support_7414.

## Results

### Patient characteristics

**Fig. 1:**
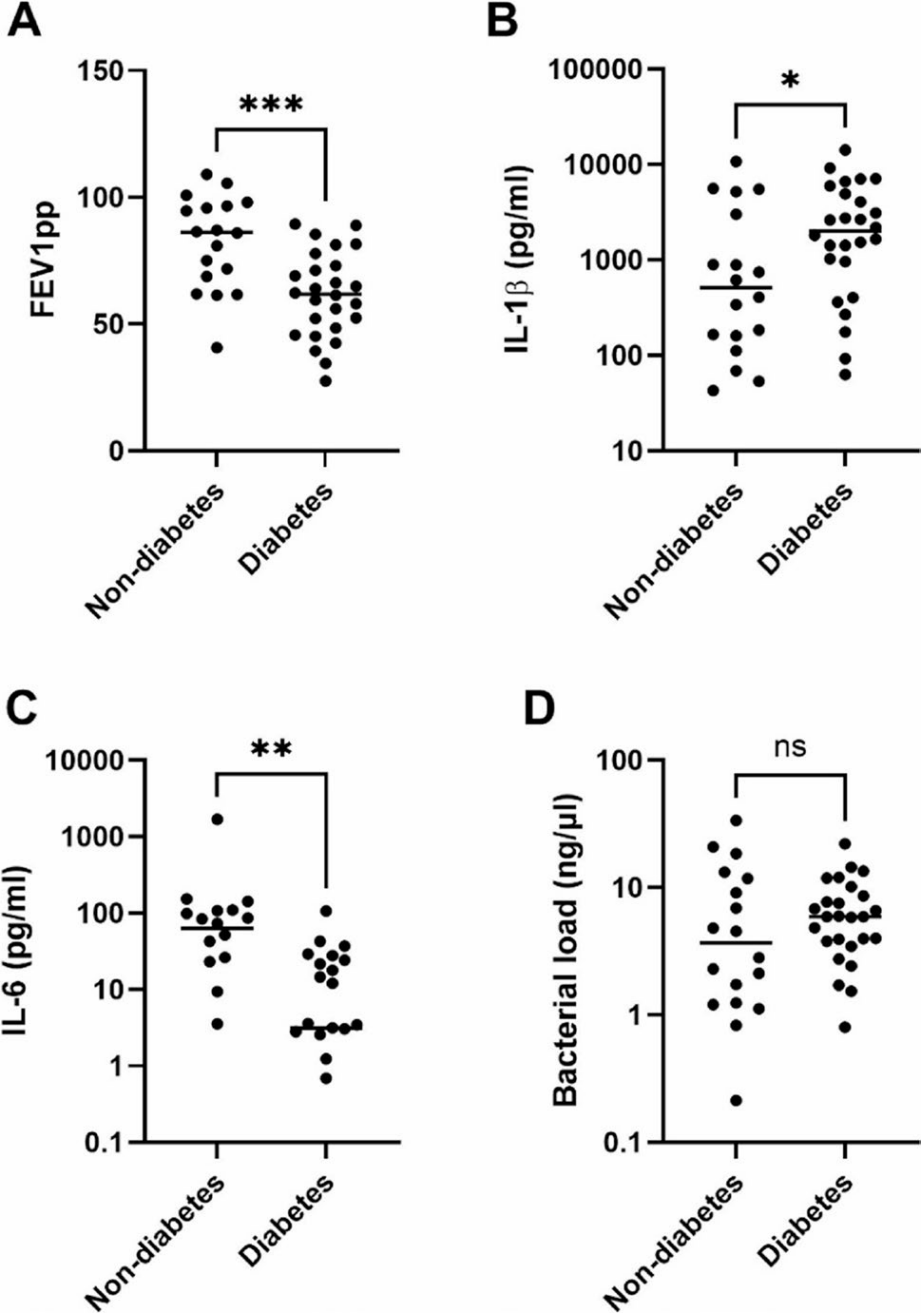
Lung function and expression of inflammatory cytokines in sputum samples of pwCF without and with CFRD. Lung function was measured as forced expiratory volume in 1 second in percent of predicted (FEV1pp) (**A**). Sputum samples from pwCF with (n=26) or without CFRD (n=18) were analysed for the inflammatory cytokines IL-1β (**B**) and IL-6 (**C**) using Mesoscale U-PLEX assays, and bacterial DNA using qPCR of the 16SDNA gene (**D**). Bars represent median values. *=p<0.05, **=p<0.01, ***=p<0.001, ns = not significant. PwCF: people with cystic fibrosis; CFRD: cystic fibrosis-related diabetes.

A total of 44 patients were enrolled in the study, of which 26 (59%) had a confirmed diagnosis of CFRD at the time of sputum collection. The CFRD group had a significantly lower lung function (*p* = 0.006) (Fig. 1A) and elevated HbA1c (*p* < 0.001) compared to individuals without CFRD (Table 1). No statistically significant differences were observed between the groups in terms of age, sex and chronic *P. aeruginosa* infection (Table 1). Eighteen of the patients with CFRD (69%) were treated with insulin.

**Table 1:** Cohort characteristics

Demographic and clinical data	All participants (*n* = 44)	Participants with CFRD (*n* = 26)	Participants without CFRD (*n* = 18)	*P*-value
Age in years median (IQR)	36 (27–40)	39 (28–42)	31 (25–39)	0.28
Female n (%)	12 (27)	7 (27)	5 (28)	0.95
Homozygous F508del n (%)	28 (64)	16 (62)	12 (67)	0.73
BMI (kg/m^2^) median (IQR)	23.1 (20.7–24.6)	22.7 (20.6–25.4)	23.2 (21–24.1)	0.98
FEV1pp median (IQR)	68.9 (56.6–86.3)	61.9 (47.7–74.3)	86.2 (67.1–97)	0.006
Chronic *P. aeruginosa* n (%)	27 (61)	16 (62)	11 (61)	0.98
Pancreatic insufficiency n (%)	42 (95)	26 (100)	16 (89)	0.08
HbA1c mmol/mol median (IQR)	41 (36.3–45.8)	44.5 (40.8–54.8)	37 (33.8–40)	< 0.001
Treatment with ETI n (%)	5 (11)	2 (7)	3 (16)	0.36

*CFRD* Cystic fibrosis-related diabetes, *BMI* Body mass index, *FEV1pp* Forced expiratory volume in 1 s in percent of predicted, *ETI* Elexacaftor-Tezacaftor-Ivacaftor

Analysis of sputum cytokine levels demonstrated significantly elevated concentrations of IL-1β (*p* = 0.039) and significantly lower IL-6 (*p* = 0.0015) in participants with CFRD (Fig. 1B-C). No significant differences were observed in sputum bacterial load between the groups (Fig. 1D).

### Lung microbial diversity

**Fig. 2:**
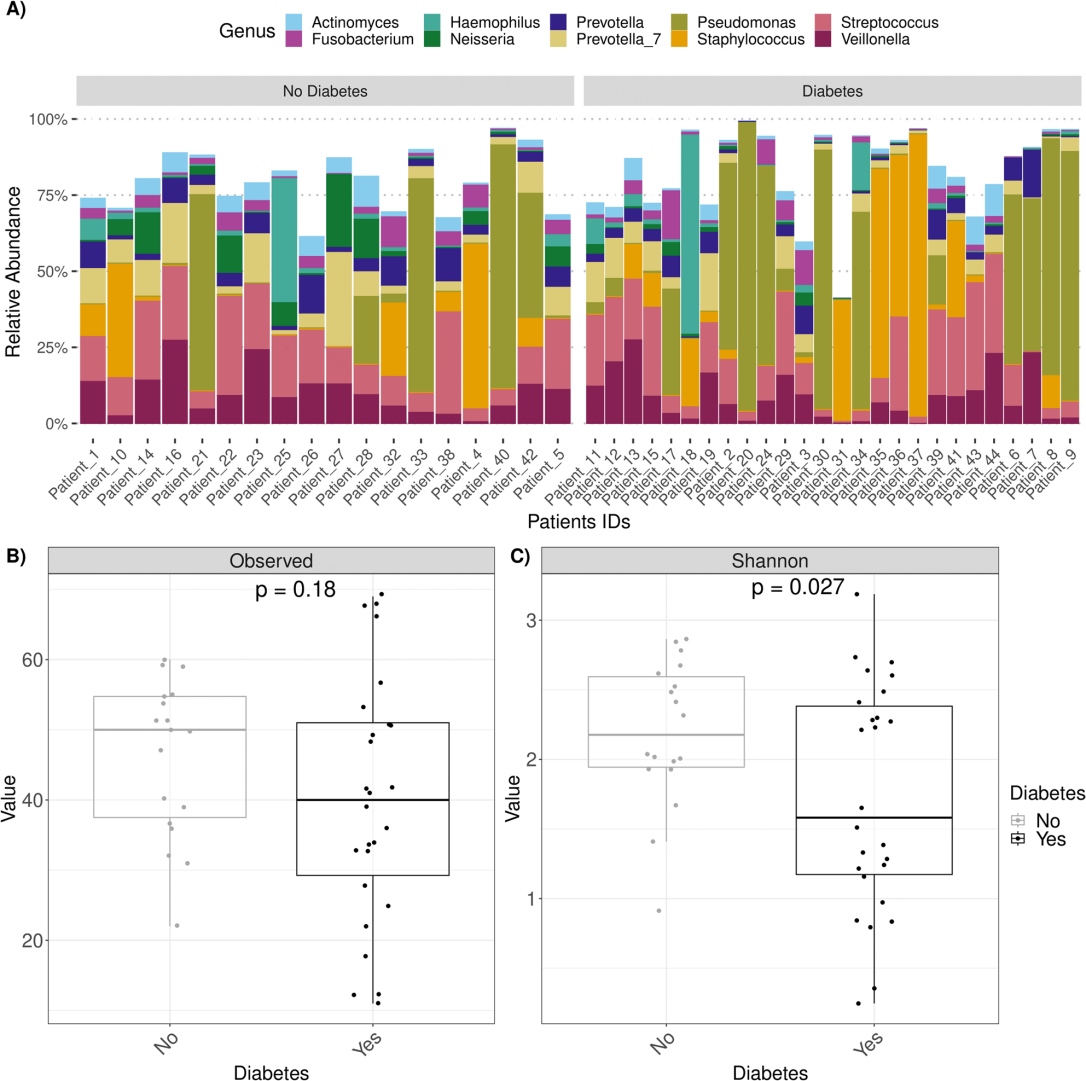
Lung microbial composition of the study samples at genus level. The figure shows the microbiome of all included study samples from each patient. The patient diabetes status (no diabetes and diabetes) is indicated above the bars, and each bar displays the relative abundance of the 10 most common bacterial genera in this study. The patient ID is indicated below each bar (**A**) Alpha diversity of the sputum samples of pwCF without and with CFRD. The microbiome composition of each sample was assessed based on species richness (number of observed genera) (**B**) and genus diversity (Shannon index) (**C**). *=*p*<0.05, **=*p*<0.01, ns = not significant. PwCF: people with cystic fibrosis; CFRD: cystic fibrosis-related diabetes

Sequencing of the 16s rRNA gene was used to analyse lung microbiome in pwCF with and without CFRD. The relative abundances of the ten most prevalent bacterial genera for individual patients in each group are presented in Fig. 2A. *Pseudomonas* and *Staphylococcus* were common genera in both groups and tended to dominate the microbiome when present.

Airway microbiomes in sputum samples were assessed using both alpha and beta diversity metrics to compare microbial diversity and composition between the CFRD and non-CFRD groups. The total number of observed genera did not differ between groups (Fig. 2B), suggesting comparable genus richness. However, the Shannon index, which accounts for both richness and evenness, was significantly lower in the CFRD group (*p* = 0.027; Fig. 2C), indicating reduced microbial diversity and a more uneven community structure within this group of samples. Consistently, unadjusted linear regression showed that CFRD was associated with lower Shannon diversity (B = -0.46, 95% CI -0.90 to -0.23, *p* = 0.039). However, after adjusting for FEV1pp, this association was no longer significant (B = 0.001, 95% CI − 0.43 to 0.44, *p* = 1.0), whereas lung function remained significantly associated with Shannon diversity (B = 0.022, 95% CI 0.012 to 0.033, *p* < 0.001). These results indicate that the reduced Shannon diversity observed in pwCF with CFRD is predominantly driven by impaired lung function, an integral component of the clinical phenotype of CFRD, rather than by CFRD itself.

**Fig. 3:**
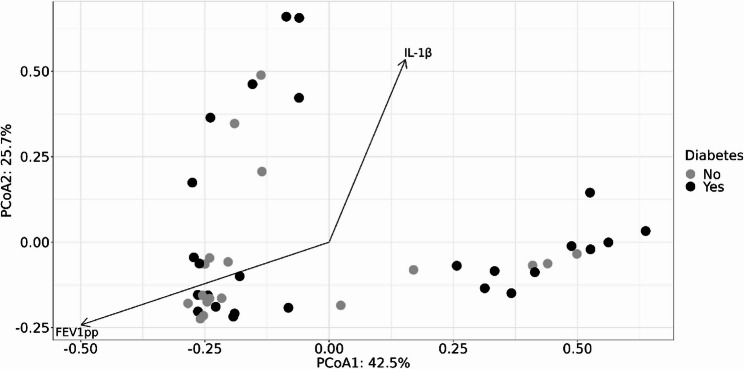
Beta diversity of the sputum samples of pwCF with and without CFRD. Principal coordinate analysis (PCoA) plot demonstrating the distance in the microbiome composition between samples classified as diabetes (black) versus no diabetes (grey), based on Bray–Curtis distance. Correlation of the ordination with clinical variables (significance tested by envfit function) is represented with the arrows of the significant clinical variables FEV1pp and IL-1β to the ordination. PwCF: people with cystic fibrosis; CFRD: cystic fibrosis-related diabetes; FEV1pp: forced expiratory volume in 1 s in percent of predicted

Beta diversity, which measures differences in microbial community composition between samples, was assessed using the Bray-Curtis dissimilarity index. Principal coordinates analysis (PCoA) did not reveal a significant clustering between the CFRD and non-CFRD groups (*p* = 0.18; Fig. 3), suggesting that CFRD status alone does not drive major shifts in overall bacterial community composition in our cohort. However, correlating beta diversity with lung function and sputum cytokines demonstrate that FEV1pp (*p* = 0.001) and sputum IL-1β levels (*p* = 0.002) were significantly associated with microbial community compositions (Fig. 3).

**Fig. 4:**
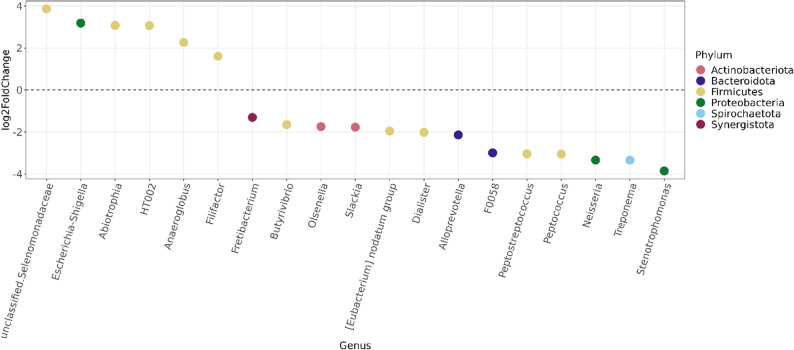
The differential abundance of lung microbiome in pwCF with and without CFRD. Differential abundance analysis was used to identify enriched microorganisms in samples graded as pwCF with and without CFRD. The figure shows genera with adjusted p-values < 0.05. Each circle represents a genus, and all genera with a log2FoldChange above zero are significantly more abundant among diabetes patients. The different colours represent different phyla. PwCF: people with cystic fibrosis; CFRD: cystic fibrosis-related diabetes

Differential abundance analysis revealed that the genera *Abiotrophia*, *Anaeroglobus* and *Escherichia-Shigella* were significantly more prevalent in the CFRD group, while *Neisseria*, *Prevotella* and *Streptococcus* were more abundant in pwCF without CFRD (Fig. 4).

## Discussion

In the present study, we compared the airway microbiome of pwCF with and without CFRD. Our findings demonstrate that the CFRD group exhibited significantly lower lung function, elevated sputum levels of IL-1ß, and decreased microbial diversity in sputum samples compared to pwCF without CFRD. These results contrast with those reported by Vasiljevs et al., who observed increased alpha diversity in the sputum microbiome of pwCF with CFRD [35]. Several methodological and cohort differences may account for these discrepancies. Notably, the prevalence of *P. aeruginosa* infection was higher in the CFRD group within the Vasiljevs cohort, whereas in our study, persistent *P. aeruginosa* colonization was comparable between groups. Furthermore, our cohort exhibited generally higher lung function, with the CFRD group showing similar FEV1pp to the non-CFRD group in Vasiljevs et al. Finally, we defined CFRD based on OGTT results in accordance with CF foundation guidelines, whereas Vasiljevs et al. relied primarily on elevated HbA1c levels [18, 35]. Notably, both studies observed significantly lower FEV1pp in the CFRD group compared to pwCF without diabetes, while age and gender distributions were similar across groups. Reduced lung function and elevated airway inflammation have previously been associated with decreased airway microbial diversity [36, 37], and our observation of lower alpha diversity in the CFRD group aligns with this relationship.

Differential abundance analysis revealed enrichment of *Escherichia/Shigella* in the airway microbiome of the CFRD group. *Escherichia/Shigella* has previously been reported to be more abundant in the gut microbiome of patients with type 2 diabetes compared to healthy controls, suggesting a potential association with diabetes [38, 39]. In contrast, the non-CFRD group exhibited a higher relative abundance of genera commonly associated with a healthier airway microbiome, including *Prevotella*, and *Streptoccocus* [10, 11]. Notably, *Prevotella* has been implicated in exerting inhibitory effects against *P. aeruginosa* biofilm formation [40], and airway microbiomes dominated by *Streptococcus* have been linked to clinical stability and less severe pulmonary disease in pwCF [41, 42]. These data may indicate that CFRD is associated with a more dysbiotic microbiome.

Beta diversity analysis did not reveal distinct clustering of microbiomes between pwCF with and without CFRD, suggesting that CFRD status alone may not drive major shifts in overall microbial community structure in our cohort. However, we identified significant correlations between beta diversity and both FEV1pp and IL-1β, indicating that lung function and inflammation may influence microbial compositions more strongly than CFRD status per se.

Given the previously reported association between reduced lung function and lower microbiome diversity [36, 37, 43] a key limitation of our study is the lower lung function observed in the CFRD group. While this reflects the clinical presentation of CFRD, it highlights the difficulty in separating microbiome alterations driven specifically by CFRD from those arising secondary to impaired lung function. In our analysis, the association between CFRD and reduced microbial diversity was largely explained by differences in FEV1pp, suggesting that lung function mediates a substantial part of the observed CFRD-microbiome relationship. Further studies with larger cohorts and longitudinal design would be required to clarify the specific contributions of CFRD to airway dysbiosis. Other factors known to influence airway microbiome composition in CF include increasing age [43] and *P. aeruginosa* infection [44], both of which are also associated with CFRD. However, neither age nor *P. aeruginosa* colonization differed between the groups in our cohort.

Additional limitations include the single centre design with a relatively small cohort size which may limit statistical power and generalizability. Even so, this is to our knowledge the largest study to date investigating the impact of CFRD on the airway microbiome. Moreover, a gender disparity was present in the total cohort, although the distribution did not differ between the two compared groups. We also acknowledge the lack of data on antibiotic treatment, which is known to affect the microbiota [45]. Given that reduced lung function may correlate with increased disease severity and the need for more frequent antibiotic treatment, this could have influenced microbiome composition. Finally, sputum samples were collected before Elexacaftor-Tezacaftor-Lumacaftor (ETI) was introduced to pwCF in Sweden. Evidence suggests that ETI can significantly alter the airway microbiome, and future studies should explore the interplay between CFRD and airway microbiome dynamics during ETI treatment [46].

## Conclusion

Our findings demonstrate that CFRD is associated with reduced lung function, elevated airway inflammation, and decreased alpha diversity in the sputum from pwCF. While CFRD status alone did not significantly alter overall microbial community composition, the enrichment of *Escherichia/Shigella* in individuals with CFRD, alongside a relative depletion of genera linked to healthier airway profiles, further suggests a shift towards a dysbiotic microbial composition. The association between CFRD and reduced alpha diversity was largely attenuated after adjustment for FEV1pp, suggesting that these microbiome differences likely reflect the broader clinical phenotype of declining pulmonary health rather than a strong independent effect of CFRD itself.

As ETI therapy becomes increasingly widespread, understanding its impact on the airway microbiome in the context of CFRD represents an important area for future research.

## Data Availability

All scripts used for the bioinformatics and statistics analyses are openly available at the following repository: https://github.com/NBISweden/support_7414.
